# Angiotensin Receptor-Neprilysin Inhibitors in Patients With Heart Failure With Reduced Ejection Fraction and Advanced Chronic Kidney Disease: A Retrospective Multi-Institutional Study

**DOI:** 10.3389/fcvm.2022.794707

**Published:** 2022-03-08

**Authors:** Fu-Chih Hsiao, Chia-Pin Lin, Chun-Chen Yu, Ying-Chang Tung, Pao-Hsien Chu

**Affiliations:** ^1^Division of Cardiology, Department of Internal Medicine, Chang Gung Memorial Hospital, Chang Gung University College of Medicine, Taipei, Taiwan; ^2^Division of Nephrology, Department of Internal Medicine, Chang Gung Memorial Hospital, Chang Gung University College of Medicine, Taipei, Taiwan

**Keywords:** heart failure with reduced ejection fraction, chronic kidney disease, end-stage renal disease, angiotensin receptor-neprilysin inhibitor, sacubitril/valsartan

## Abstract

**Background:**

Data regarding using angiotensin receptor-neprilysin inhibitor (ARNI) in patients with both heart failure with reduced ejection fraction (HFrEF) and advanced chronic kidney disease (CKD) are limited.

**Methods and Results:**

Between January 2016 and December 2018, patients with HFrEF and advanced CKD (estimated glomerular filtration rate [eGFR] ≤ 30 mL/min/1.73 m^2^) were identified from a multi-institutional database in Taiwan. Patients who had never been prescribed with an ARNI, angiotensin-converting enzyme inhibitor (ACEI), or angiotensin receptor blocker (ARB) were excluded. We used inverse probability of treatment weighting (IPTW) to balance baseline covariates, and compared outcomes between ARNI and ACEI/ARB users. There were 206 patients in the ARNI group and 833 patients in the ACEI/ARB group. After IPTW adjustment, the mean ages (65.1 vs. 66.6 years), male patients (68.3 vs. 67.9%), left ventricular ejection fraction (30.5 vs.31.2%), eGFR (20.9 vs. 20.3 mL/min/1.73 m^2^) were comparable in the ARNI and ACEI/ARB groups. Over 85% of the patients had beta-blockers prescriptions in both groups (86.2 vs. 85.5%). After IPTW adjustment, the mean follow-up durations were 7.3 months and 6.6 months in the ARNI and ACEI/ARB groups, respectively. ARNI and ACEI/ARB users had a comparable risk of the composite clinical event (all-cause mortality or heart failure hospitalization) (hazard ratio [HR], 1.31; 95% confidence interval (CI) 0.91–1.88) and progression to dialysis (HR 1.04; 95% CI 0.54–2.03). In subgroup analysis, dialysis patients who used ARNIs were associated with higher incidence of heart failure hospitalization (subdistribution HR, 1.97; 95% CI 1.36–2.85).

**Conclusions:**

Compared with ACEIs or ARBs, ARNIs were associated with comparable clinical and renal outcomes in patients with HFrEF and advanced CKD (eGFR ≤ 30 mL/min/1.73 m^2^). In short-term, HF hospitalization may occur more frequently among ARNI users, especially in patients on dialysis.

## Introduction

Chronic kidney disease (CKD) is not uncommon in patients with heart failure with reduced ejection fraction (HFrEF), as they have similar upstream risk factors and interact to increase adverse events. Reduced estimated glomerular filtration rate (eGFR) has been reported to be an independent predictor of mortality and hospitalization in patients with heart failure (HF) (1). In addition, HF patients have been shown to have a 2-fold faster decline in eGFR than the general population (2). Although the number of patients with both advanced CKD (eGFR ≤ 30 mL/min/1.73 m^2^) and HFrEF is increasing globally with high morbidity and mortality, (3, 4) they have been systemically excluded from randomized trials of pharmacological therapies for HFrEF. Thus, evidence-based therapies for this special population are still lacking.

Sacubitril/valsartan, an angiotensin receptor-neprilysin inhibitor (ARNI), was added to guidelines for the treatment of HFrEF after the publication of the Prospective comparison of Angiotensin Receptor-neprilysin inhibitor (ARNI) with Angiotensin converting enzyme inhibitor (ACEI) to Determine Impact on Global Mortality and morbidity in Heart Failure (PARADIGM-HF) trial (5) In subgroup analysis, sacubitril/valsartan were found to be superior to enalapril in reducing cardiovascular mortality or HF hospitalization, irrespective of the presence or absence of CKD. However, patients with eGFR below 30 mL/min/1.73 m^2^ were again not enrolled in this trial. The instructions for users of sacubitril/valsartan in Taiwan do not list advanced CKD as a contraindication. Thus, despite a lack of evidence, some cardiologists in Taiwan prescribed sacubitril/valsartan for patients with HFrEF and advanced CKD in an attempt to either improve symptoms, reduce HF hospitalization, or prolong survival.

Using a multi-institutional claims database, the purpose of the present study was to report the baseline characteristics and pharmacological therapies of patients with both HFrEF and advanced CKD from real-world experience. In addition, the clinical, renal, and echocardiographic outcomes of patients receiving ARNIs were compared to those receiving angiotensin-converting enzyme inhibitors (ACEIs) or angiotensin receptor blockers (ARBs).

## Methods

### Database

Data for the present study were obtained from the Chang Gung Research Database (CGRD). The CGRD contains the standardized electronic medical records from seven institutes of Chang Gung Memorial Hospital (CGMH), which is the largest hospital system in Taiwan with 10,070 beds and admits more than 280,000 patients each year. The outpatient department visits and emergency department visits to CGMH were over 8,500,000 and 500,000, respectively in 2015. CGRD has collected and standardized the electronic medical records of all patients since 2000 without selection criteria. One strength of the CGRD is that it includes each patient's medical diagnosis, laboratory results, image findings, medications, and procedure reports. Diagnoses were registered using International Classification of Diseases, 9th Revision, Clinical Modification (ICD-9-CM) codes before 2016, and ICD-10 codes thereafter. More details about the CGRD have been reported elsewhere (5, 6).

The personal information of each patient was de-identified using a consistent encryption procedure; therefore, the need for informed consent was waived for this study. This study conformed to the ethical guidelines of the 1975 Declaration of Helsinki and was approved by the Institutional Review Board of CGMH, Linkou (IRB number: 202000410B0).

### Study Design

**Figure 1:**
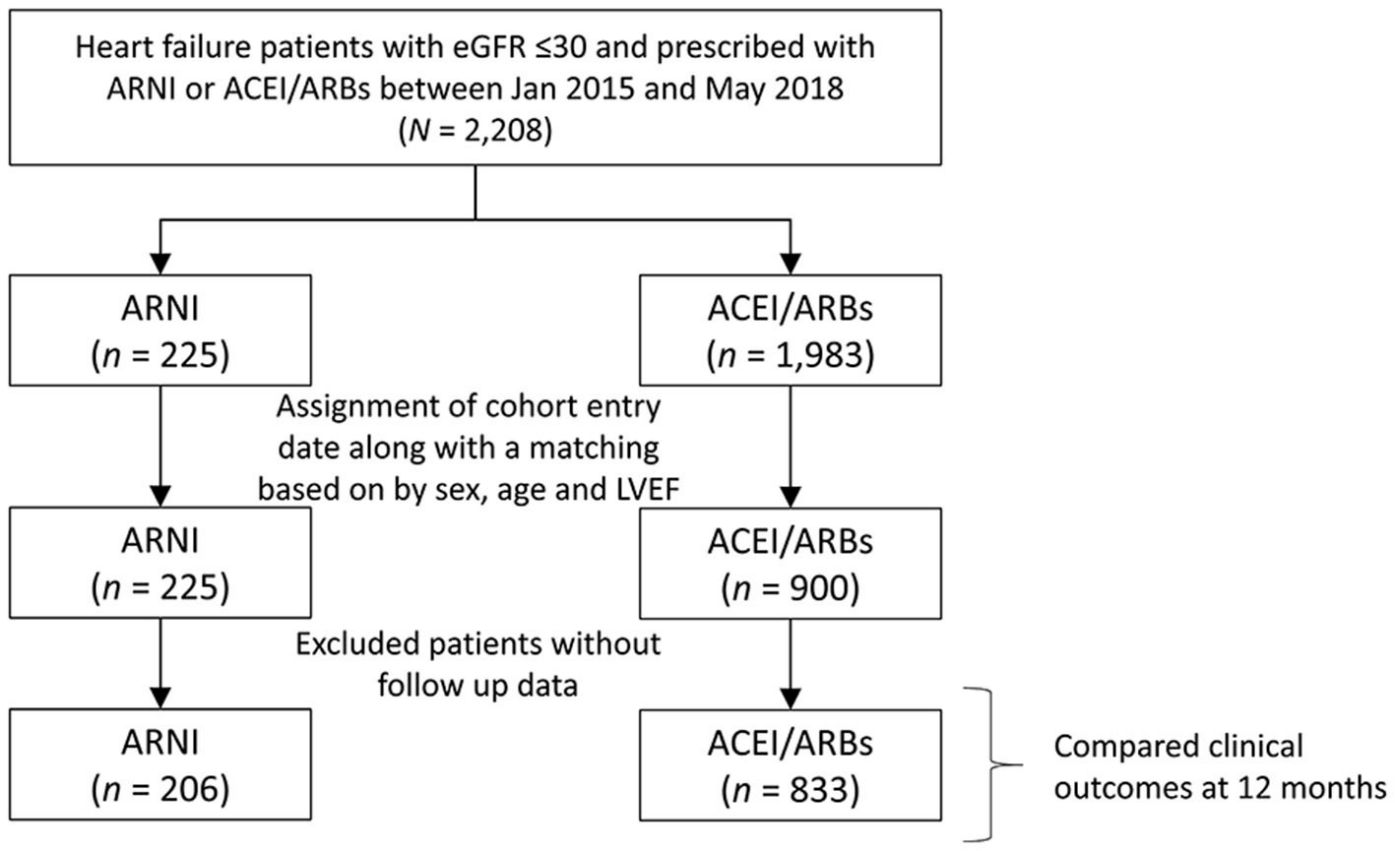
Flowchart of the inclusion and exclusion criteria of the study patients.

Figure 1 shows the process of patient inclusion and exclusion. Between January 2016 and December 2018, patients with both HFrEF and advanced CKD were identified from the CGRD. Patients with HFrEF had to fulfill the following two criteria: (1) a principal or secondary diagnosis of HF in inpatient or outpatient claims data; (2) a baseline left ventricular ejection fraction (LVEF) less than 40% by echocardiography within 3 months before the first diagnosis of HF. The identification of patients with HFrEF in the CGRD has been reported previously (7, 8). Advanced CKD was defined as two consecutive records of eGFR ≤ 30 mL/min/1.73 m^2^ in the previous year before the cohort entry date (defined later).

HFrEF patients who had been prescribed an ARNI, ACEI, or ARB (candesartan, valsartan, losartan, or a fixed-dose combinations including these three ARBs) for at least 30 days were further identified. The date of the first prescription of an ARNI was defined as the cohort entry date for the ARNI group. The cohort entry date for the ACEI/ARB group was assigned from the ARNI group to avoid immortal time bias (9). In the meanwhile, the two groups were frequency matched based on age, sex, and baseline LVEF. The baseline period was defined as the 12 months before the cohort entry date. We excluded those who had no serum creatinine data and those with an eGFR > 30 mL/min/1.73 m^2^. Patients without follow-up data were also excluded.

### Covariates

Data on covariates including baseline characteristics (age, sex, height, and weight), vital signs, previous cardiac treatments, comorbidities, medications, laboratory, and echocardiographic findings were extracted from the CGRD. Body height, body weight, blood pressure, and heart rate were obtained from a vital sign sub-database within 3 months before the cohort entry date. Comorbidities were defined if any inpatient or two outpatient diagnoses were recorded with ICD-9 or ICD-10 codes during the baseline period. Data on prior cardiac treatments, including valve surgery, cardiac resynchronization therapy and coronary artery bypass graft were extracted from inpatient data. Medications, laboratory, and echocardiographic results were obtained within 3 months before the cohort entry date.

### Outcomes

The clinical outcomes of interest were all-cause mortality, HF hospitalization, the composite of both, and admission due to any cause. After excluding patients on dialysis at baseline, the renal outcomes observed were progression to end-stage renal disease (ESRD) and severe hyperkalemia (serum potassium ≥ 6 mEq/L). HF hospitalization was defined as having a principal discharge diagnosis of HF and at least one treatment during hospitalization, including diuretics, nitrites, or inotropic agents. Progression to ESRD was defined as maintenance dialysis for ≥ 28 days. The follow-up period was defined as the period from the cohort entry date until the first occurrence of an outcome, day of mortality, the last outpatient visits or discharge date in the CGRD, the end of the study period (December 31, 2018), or at 12th month, whichever occurred first.

Finally, changes in echocardiographic parameters (mean LVEF, left ventricular end diastolic and end systolic diameters, left atrial diameter) from baseline in each group will be compared, using persons with available follow-up echocardiography after the index date.

### Statistical Analysis

To achieve comparability in clinical outcomes between the study groups, we conducted inverse-probability-of-treatment weighting (IPTW) based on propensity score. Compared to propensity score matching (PSM), the results based on IPTW have greater statistical power without losing sample size. The propensity score was calculated using multivariable logistic regression where the study group was regressed on all of the covariates (listed in Table 1, except the follow-up month) and possible interactions among the covariates were not considered. To reduce the impact of extreme propensity scores, we used a stabilized weight (10). We used the total cohort and compared the risk of all-cause mortality, HF hospitalization, and admission due to any cause after IPTW adjustment. To compare the risk of progression to ESRD and severe hyperkalemia, we performed another IPTW adjustment after excluding persons on dialysis at baseline. The balance of covariate distribution between groups was checked using the absolute value of the standardized difference (STD) before and after weighting, where a value of <0.2 was considered to be a small difference. In addition, due to the existence of missing laboratory data, the missing values were first imputed using the single expectation–maximization imputation method, and IPTW was conducted using the imputed data.

**Table 1:** Baseline characteristics between the ARNI and ACEI/ARB groups before and after IPTW adjustment.

	Before EM imputation and IPTW^*^					**After EM imputation and IPTW** ^†^		
**Variable**	**Valid** ***N***	**Total** **(*n* = 1,039)**	**ARNI** **(*n* = 206)**	**ACEI/ARB** **(*n* = 833)**	**STD**	**ARNI** **(*n* = 974.8)**	**ACEI/ARB** **(*n* = 1,044.1)**	**STD**
**Demographics**								
Age, years	1,039	66.4 ± 13.3	65.1 ± 14.4	66.8 ± 13.0	−0.12	65.1 ± 16.0	66.6 ± 12.9	−0.11
Male	1,039	701 (67.5)	142 (68.9)	559 (67.1)	0.04	68.3%	67.9%	0.01
Height cm	875	161.1 ± 11.0	162.5 ± 9.4	160.8 ± 11.3	0.17	162.4 ± 9.8	161.1 ± 10.9	0.13
Body weight kg	971	62.4 ± 13.0	65.0 ± 14.4	61.8 ± 12.6	0.24	64.4 ± 14.9	62.2 ± 12.7	0.16
CKD group	1,039							
Stage 4 (eGFR: 15–30)		325 (31.3)	80 (38.8)	245 (29.4)	0.20	39.5%	32.0%	0.16
Stage 5 (eGFR <15)		96 (9.2)	16 (7.8)	80 (9.6)	−0.07	6.0%	8.9%	−0.11
ESRD on dialysis		618 (59.5)	110 (53.4)	508 (61.0)	−0.15	54.5%	59.1%	−0.09
**Vital signs**								
SBP mmHg	1,037	129.6 ± 24.0	129.3 ± 23.6	129.7 ± 24.1	−0.02	128.0 ± 23.7	129.3 ± 23.8	−0.06
DBP mmHg	1,037	72.0 ± 16.8	73.0 ± 17.2	71.8 ± 16.7	0.07	70.9 ± 16.8	71.9 ± 16.4	−0.06
Heart rate	1,029	79.9 ± 16.3	79.3 ± 16.1	80.1 ± 16.4	−0.05	80.1 ± 14.9	79.9 ± 16.2	0.01
**History of cardiac treatment**								
Valve surgery	1,039	39 (3.8)	8 (3.9)	31 (3.7)	0.01	5.0%	3.6%	0.07
CRT	1,039	17 (1.6)	5 (2.4)	12 (1.4)	0.07	2.6%	1.5%	0.07
CABG	1,039	95 (9.1)	28 (13.6)	67 (8.0)	0.18	8.5%	8.8%	−0.01
**Comorbidities**								
Coronary artery disease	1,039	653 (62.8)	140 (68.0)	513 (61.6)	0.13	61.7%	63.0%	−0.03
Myocardial infarction	1,039	319 (30.7)	66 (32.0)	253 (30.4)	0.04	28.1%	30.3%	−0.05
Hypertension	1,039	855 (82.3)	173 (84.0)	682 (81.9)	0.06	84.5%	81.7%	0.07
Dyslipidemia	1,039	584 (56.2)	121 (58.7)	463 (55.6)	0.06	49.8%	55.9%	−0.12
Diabetes mellitus	1,039	625 (60.2)	127 (61.7)	498 (59.8)	0.04	58.9%	59.7%	−0.02
Stroke	1,039	117 (11.3)	17 (8.3)	100 (12.0)	−0.12	11.3%	11.2%	0.00
Atrial fibrillation	1,039	177 (17.0)	38 (18.4)	139 (16.7)	0.05	18.5%	17.1%	0.04
Chronic obstructive pulmonary disease	1,039	107 (10.3)	24 (11.7)	83 (10.0)	0.05	11.3%	10.6%	0.02
Peripheral arterial disease	1,039	126 (12.1)	22 (10.7)	104 (12.5)	−0.06	11.2%	12.5%	−0.04
**Medications**								
Beta–blockers	1,039	892 (85.9)	183 (88.8)	709 (85.1)	0.11	86.2%	85.5%	0.02
MRAs	1,039	228 (21.9)	63 (30.6)	165 (19.8)	0.25	31.3%	22.8%	0.19
Ivabradine	1,039	139 (13.4)	55 (26.7)	84 (10.1)	0.44	13.7%	13.8%	0.00
Loop diuretics	1,039	669 (64.4)	155 (75.2)	514 (61.7)	0.29	70.9%	64.6%	0.14
Digoxin	1,039	147 (14.1)	34 (16.5)	113 (13.6)	0.08	16.0%	14.5%	0.04
Amiodarone	1,039	132 (12.7)	37 (18.0)	95 (11.4)	0.19	17.1%	13.1%	0.11
**Laboratory data**								
Creatinine mg/dL^‡^	421	3.3 ± 1.8	3.2 ± 1.8	3.4 ± 1.8	−0.13	3.2 ± 1.7	3.3 ± 1.7	−0.02
eGFR mL/min/1.73 m^2^^‡^	421	20.1 ± 6.8	21.5 ± 6.7	19.7 ± 6.8	0.27	20.9 ± 6.3	20.3 ± 6.7	0.08
BNP pg/mL	720	2,160 [838, 4442]	2,130 [669, 4700]	2,209 [888, 4320]	NA	2,130 [974, 4675]	2,216 [888, 4426]	NA
BUN mg/dL	1,006	57.6 ± 27.8	56.8 ± 27.4	57.8 ± 28.0	−0.04	55.7 ± 26.5	57.0 ± 27.6	−0.05
Sodium (Na) mEq/L	1,017	137.2 ± 4.7	137.8 ± 5.1	137.1 ± 4.5	0.15	137.3 ± 5.0	137.2 ± 4.5	0.03
Potassium (K) mEq/L	1,028	4.3 ± 0.7	4.3 ± 0.8	4.4 ± 0.7	−0.08	4.4 ± 0.8	4.3 ± 0.7	0.02
Uric acid mg/dL	862	7.2 ± 2.6	7.3 ± 2.8	7.1 ± 2.5	0.08	7.5 ± 2.8	7.2 ± 2.6	0.14
Calcium mg/dL	901	8.9 ± 0.9	8.9 ± 0.8	8.9 ± 0.9	0.01	8.9 ± 0.8	8.9 ± 0.9	0.01
Phosphates mg/dL	841	4.8 ± 1.7	4.7 ± 1.7	4.8 ± 1.7	−0.05	4.6 ± 1.8	4.8 ± 1.7	−0.09
Hemoglobin g/dL	1,023	10.6 ± 2.0	11.0 ± 2.1	10.5 ± 1.9	0.28	10.7 ± 1.7	10.6 ± 2.0	0.05
Hematocrit g/dL	1,022	32.2 ± 5.9	33.7 ± 6.2	31.9 ± 5.7	0.31	32.7 ± 5.2	32.3 ± 5.9	0.07
Serum albumin mg/dL	881	3.5 ± 0.5	3.6 ± 0.5	3.5 ± 0.5	0.11	3.6 ± 0.5	3.5 ± 0.5	0.17
Proteinuria (U/A dipstick) mg/dL	625							
Negative (0–4)		78 (7.5)	23 (11.2)	55 (6.6)	0.16	10.3%	8.1%	0.07
Trace (5–29)		42 (4.0)	10 (4.9)	32 (3.8)	0.05	4.8%	4.2%	0.03
≥1+ (≥30)		505 (48.6)	86 (41.7)	419 (50.3)	−0.17	50.9%	48.6%	0.05
Unknown		414 (39.8)	87 (42.2)	327 (39.3)	0.06	34.0%	39.1%	−0.11
**Echocardiography**								
LVEF%	1,039	31.3 ± 6.7	28.7 ± 6.9	32.0 ± 6.5	−0.49	30.5 ± 6.9	31.2 ± 6.9	−0.10
LVEDD mm	1,036	57.4 ± 8.5	60.0 ± 8.7	56.8 ± 8.3	0.38	58.6 ± 8.5	57.5 ± 8.4	0.13
LVESD mm	1,036	46.4 ± 9.4	50.7 ± 8.6	45.4 ± 9.3	0.60	47.6 ± 8.6	46.5 ± 9.5	0.12
LA mm	1,034	44.1 ± 7.7	45.4 ± 7.6	43.8 ± 7.7	0.22	45.2 ± 7.4	44.1 ± 7.6	0.14
MR severity	1,002							
Severe		88 (8.8)	25 (12.6)	63 (7.8)	0.16	11.1%	9.6%	0.05
Moderate		258 (25.7)	50 (25.1)	208 (25.9)	−0.02	27.1%	25.6%	0.03
Mild		537 (53.6)	97 (48.7)	440 (54.8)	−0.12	51.2%	53.3%	−0.04
Trivial/None		119 (11.9)	27 (13.6)	92 (11.5)	0.06	10.5%	11.5%	−0.03
Follow up month	1,039	6.9 ± 4.2	7.4 ± 4.1	6.7 ± 4.2	0.17	7.3 ± 4.2	6.6 ± 4.2	0.17

*EM, expectation maximization; IPTW, inverse probability of treatment weighting; ARNI, angiotensin receptor–neprilysin inhibitor; ACEI, angiotensin–converting enzyme inhibitor; ARB, angiotensin receptor blocker; STD, standardized difference; CKD, chronic kidney disease; eGFR, estimated glomerular filtration rate; ESRD, end–stage renal disease; SBP, systolic blood pressure; DBP, diastolic blood pressure; CRT, cardiac resynchronization therapy; CABG, coronary artery bypass graft; MRA, mineralocorticoid receptor antagonist; BUN, blood urea nitrogen; BNP, B–type natriuretic peptide; LVEF, center ventricular ejection fraction; LVEDD, center ventricular end–diastolic dimension; LVESD, center ventricular end–systolic diameter; LA, center atrium; MR, mitral regurgitation; NA, not available*.

* *Data are presented as number (%), mean ± standard deviation or median [25th, 75th percentile];*

† *Data are presented as %, mean ± standard deviation or median [25th, 75th percentile];*

‡ *Patients with dialysis at baseline were excluded*.

The risks of fatal outcomes (i.e., composite of all-cause death and HF hospitalization, all-cause death, MAKEs) between groups were compared using a Cox proportional hazard model. The incidence of other non-fatal time-to-event outcomes (i.e., HF hospitalization, progression to ESRD) between groups was compared using a Fine and Gray sub-distribution hazard model which considered all-cause death during follow-up as a competing risk. We further conducted subgroup analysis stratified by renal function status (non-dialysis vs. dialysis) on clinical events including the composite of all-cause death or HF hospitalization, HF hospitalization, and all-cause death. Finally, changes in echocardiography data from baseline to the 12th month within either group were compared using the paired sample *t*-test for continuous variables and the McNemar test for dichotomized variables (severe mitral regurgitation). Differences in changes between the ARNI and ACEI/ARB groups were compared using generalized estimating equations in which the interaction of ‘group by time point' was included in the model.

A two-sided *P*-value of < 0.05 was considered to be statistically significant. All statistical analyses were performed using SAS Version 9.4 (SAS Institute, Cary, NC, USA).

## Results

### Baseline Characteristics

Between January 2016 and December 2018, a total of 1,039 HFrEF patients with two consecutive records of eGFR ≤ 30 mL/min/1.73 m^2^ at baseline, who received an ARNI, ACEI, or ARB, and had available follow-up information were eligible for analysis. Of these patients, 206 received ARNI and 833 patients received ACEIs or ARBs. After excluding those with ESRD at baseline, there were 96 patients in the ARNI group and 325 patients in the ACEI/ARB group.

Baseline characteristics, laboratory and echocardiographic data, and medical therapies for HFrEF before and after imputation and weighting are presented in Table 1. After IPTW, the mean ages were 65.1 ± 16.0 and 66.6 ± 12.9 years (STD = −0.11) and male patients accounted for 68.3 and 67.9% (STD = 0.05) in the ARNI and ACEI/ARB group, respectively. The prevalence of comorbidities was not substantially different between the two groups before and after weighting. Ischemic cardiomyopathy was assumed to be the most prevalent etiology for HFrEF since more than 60% of the patients had coronary artery disease and around 30% had a history of myocardial infarction in both groups after weighting. The proportion of patients with diabetes mellitus (DM) was exceptionally high (nearly 60%) in both groups after weighting.

After IPTW adjustment, LVEF (30.5 vs. 31.2%) and eGFR (20.9 vs. 20.3 mL/min/1.73 m2) were comparable in the ARNI and ACEI/ARB group, respectively. Other laboratory and echocardiographic data were not substantially different (absolute STD values <0.2). Baseline B-type natriuretic peptide were available in about 70% of the patients in both groups, and the level was high (over 2,000 pg/mL) and comparable before and after adjustment.

Beta-blockers were prescribed in more than 80% of the patients in both groups. Before weighting, the ARNI users were more likely to have a concomitant prescription of mineralocorticoid receptor blockers, ivabradine, or loop diuretics. After weighting, only mineralocorticoid receptor blockers (31.3 vs. 22.8%, STD = 0.19) were more frequently prescribed in the ARNI users.

The mean follow-up durations were 7.3 ± 4.2 months and 6.6 ± 4.2 months in the ARNI and ACEI/ARB groups, respectively (Table 1).

### Clinical Outcomes

**Figure 2:**
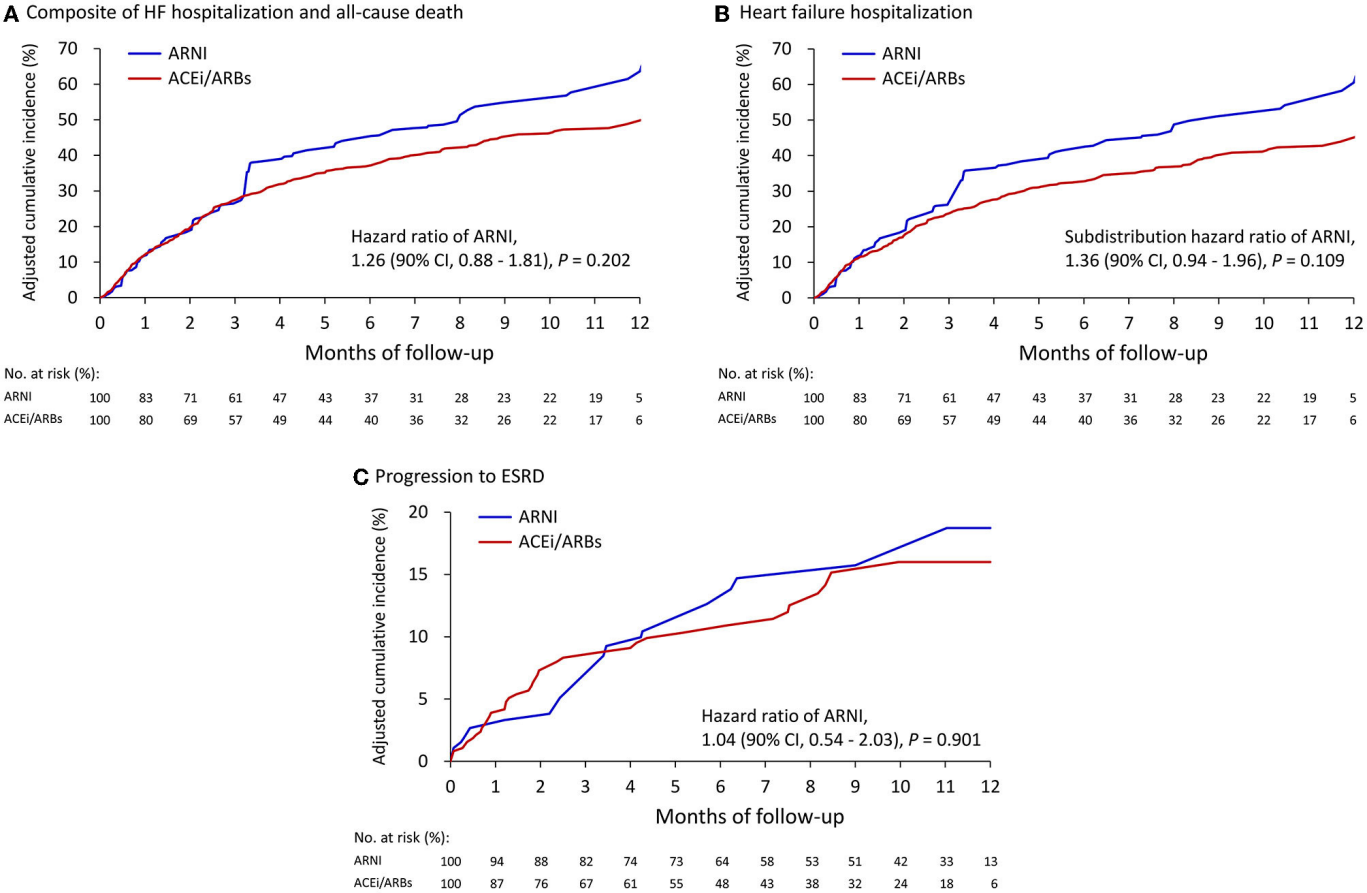
Cumulative event rate of the composite of HF hospitalization and all-cause death **(A)**, HF hospitalization **(B)**, and progression to ESRD **(C)** between the ARNI and ACEI/ARB users. **(A)** and **(B)** compared with the IPTW cohort from all patients; **(C)** after excluding persons on dialysis at baseline then creating another IPTW cohort.

Table 2 summarizes the clinical outcomes after weighting adjustments. The composite clinical outcomes (all-cause death or HF hospitalization) occurred in 47.1% of the ARNI group and 37.4% of the ACEI/ARB group (hazard ratio [HR], 1.26; 95% confidence interval (CI) 0.88–1.81) (Figure 2A). All-cause death was high and comparable between the ARNI (15.0%) and ACEI/ARB (12.9%) groups (HR, 1.03; 95% CI 0.57–1.86). There was a trend of increased HF hospitalization in the ARNI group (43.5%) compared to the ACEI/ARB group (32.2%) (subdistribution HR [SHR], 1.36; 95% CI, 0.94–1.96), although this was not significant (Figure 2B). More than half of the patients were admitted for any cause during follow up in both groups, which was comparable (SHR, 1.16; 95% CI, 0.82–1.66). Supplementary Table 1 shows the results based on matching which were consistent to that of the primary analysis. Supplementary Table 2 shows the clinical outcomes after excluding patients on dialysis at baseline and adjusting by IPTW. The results in this subgroup were similar to those of the whole cohort.

**Table 2:** Follow–up outcomes between the ARNI and ACEI/ARBs groups at 12 months of follow–up after IPTW adjustment.

	**Data before IPTW**		**Data after IPTW**			
	**ARNI** **(*n* = 206)**	**ACEI/ARB** **(*n* = 833)**	**ARNI** **(*n* = 974.8)**	**ACEI/ARB** **(*n* = 1,044.1)**	**ARNI** ***vs*. ACEI/ARB**	
**Outcome variable**					**HR/SHR (95% CI)**	***P*-value**
Primary outcome: composite of heart failure hospitalization and all–cause death	94 (45.6)	308 (37.0)	47.1%	37.4%	1.26 (0.88, 1.81)	0.202
Secondary outcome						
All–cause death	20 (9.7)	106 (12.7)	15.0%	12.9%	1.03 (0.57, 1.86)	0.935
Heart failure hospitalization	88 (42.7)	263 (31.6)	43.5%	32.2%	1.36 (0.94, 1.96)	0.109
Admission due to any cause	120 (58.3)	425 (51.0)	60.0%	50.6%	1.16 (0.82, 1.66)	0.400
Progression to ESRD (*n* = 421)^*^	17 (17.7)	45 (13.8)	14.7%	12.2%	1.04 (0.54, 2.03)	0.901
K ≥ 6 mg/dL (*n* = 421)^*^	17 (17.7)	41 (12.6)	20.3%	11.4%	1.50 (0.73, 3.05)	0.268

*IPTW, inverse probability of treatment weighting; ARNI, angiotensin receptor–neprilysin inhibitor; ACEI, angiotensin–converting enzyme inhibitor; ARB, angiotensin receptor blocker; HR, hazard ratio; SHR, subdistribution hazard ratio; CI, confidence interval; ESRD, end–stage renal disease*.

* *After excluding patients with dialysis at baseline and the IPTW was re-performed*.

### Progression to ESRD and Severe Hyperkalemia

As shown in Table 2, after excluding persons on dialysis at baseline the adjusted by IPTW, 14.7% of the patients in the ARNI group and 12.2% in the ACEI/ARB group had progressed to ESRD (SHR, 1.04; 95% CI, 0.54–2.03) (Figure 2C). Severe hyperkalemia tended to occur more frequently in the ARNI users, however the difference was not significant. Supplementary Table 1 shows the results based on matching which were consistent to that of the primary analysis.

### Clinical Outcomes Stratified by Renal Function at Baseline

**Figure 3:**
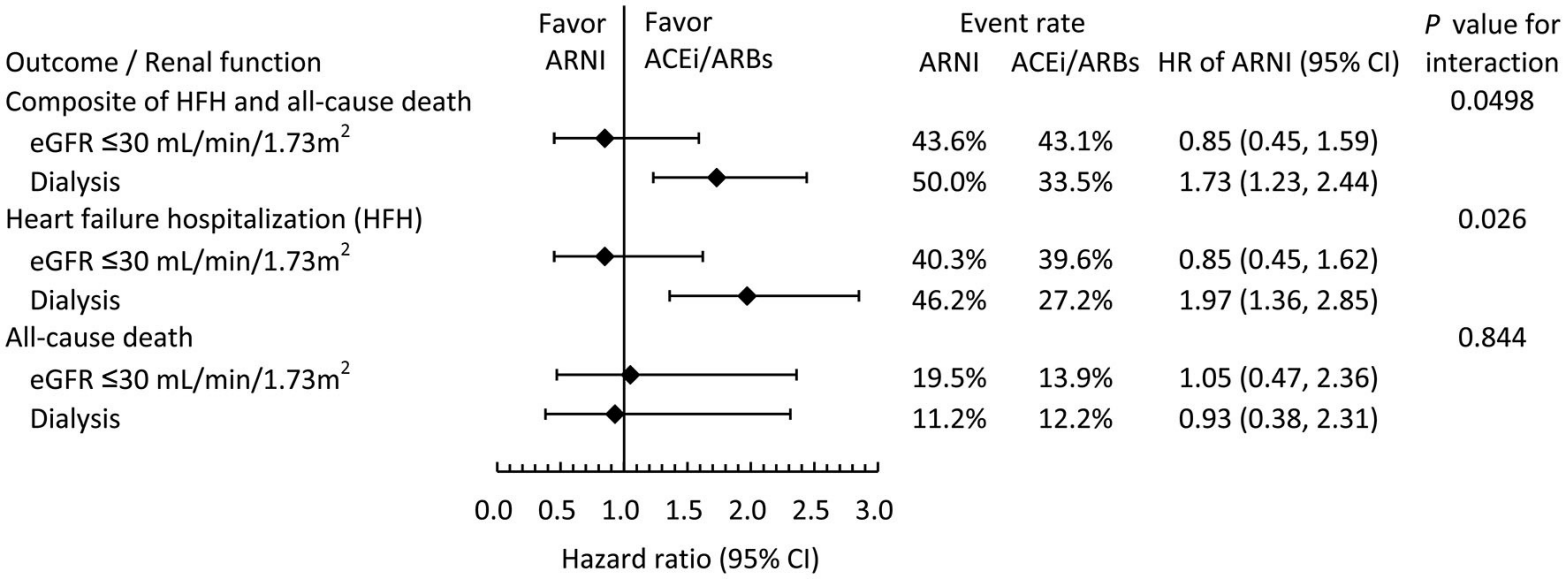
Subgroup analysis comparing the risk of clinical outcomes between the ARNI and ACEI/ARB users in the IPTW-adjusted cohort stratified by baseline renal function.

Figure 3 illustrates the subgroup analysis of clinical outcomes stratified by renal function at baseline. The results suggested that renal function at baseline significantly modified the association between the use of ARNIs and the risk of clinical outcomes, especially on the composite outcome (*P* for interaction = 0.0498) and HF hospitalization (*P* for interaction = 0.026). In the patients not receiving hemodialysis, the clinical outcomes were comparable between the ARNI and ACEI/ARB groups. However, in the patients on dialysis at baseline, the ARNI users tended to have a higher risk of the composite clinical outcome, which was driven by an elevated risk of HF hospitalization.

### Clinical Outcomes Stratified by Diabetes Mellitus at Baseline

**Figure 4:**
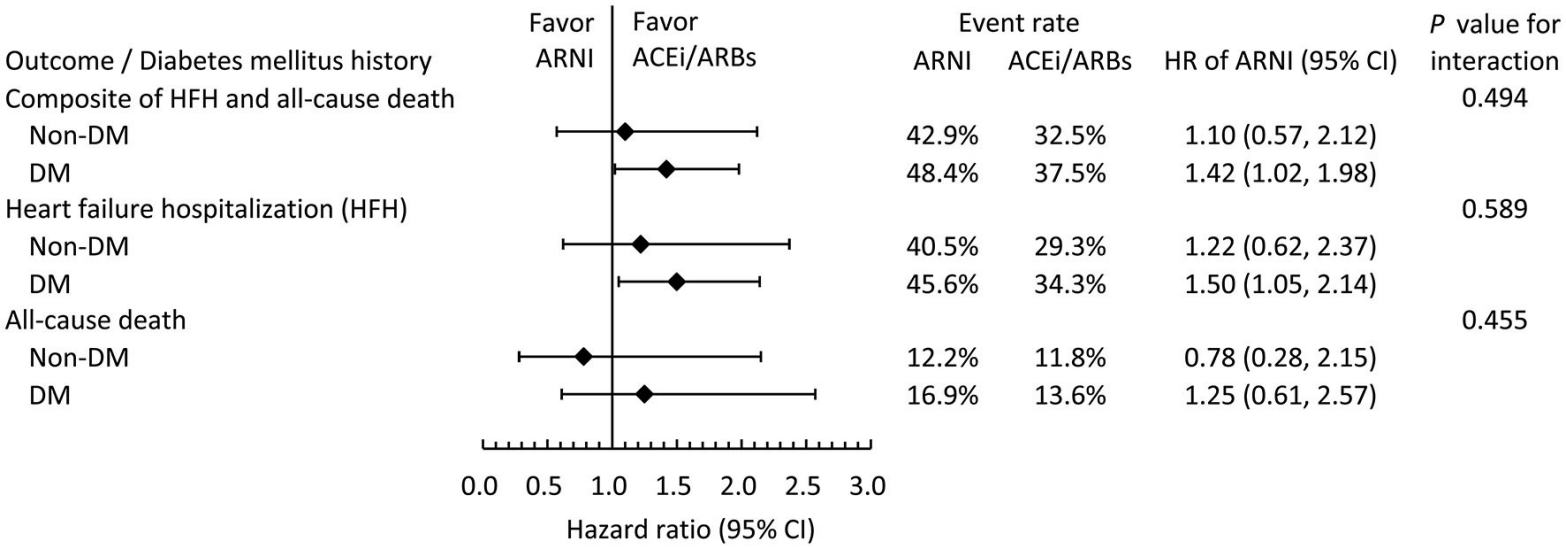
Subgroup analysis comparing the risk of clinical outcomes between the ARNI and ACEI/ARB users in the IPTW-adjusted cohort stratified by baseline DM status.

Figure 4 showed the subgroup analysis of clinical outcomes stratified by DM status at baseline. All clinical outcomes were comparable between ARNI users and ACEI/ARB users, irrespective of the presence or absence of DM.

### Echocardiographic Outcomes

Follow-up echocardiography data were available for half of the patients in both groups. Table 3 shows the changes in echocardiographic data from baseline in these patients. Reverse remodeling was observed in both groups, as evidenced by a significant increase in LVEF (change in value: 8.3 ± 14.6 vs. 10.8 ± 15.2%, *P* for interaction = 0.228) and decreases in left ventricular end-diastolic diameter (LVEDD) and left ventricular end-systolic diameter (LVESD). Compared to the ACEI/ARB users, the ARNI users had a significantly more pronounced reduction in both LVEDD (change in value: −3.1 ± 7.8 mm vs. −1.0 ± 6.1 mm, *P* for interaction = 0.013) and LVESD (change in value: >4.7 ± 9.7 mm vs. −2.1 ± 8.1 mm, *P* for interaction = 0.017). The percentage of severe mitral regurgitation remained the same from baseline to the 12th month in the ACEI/ARB group. In the ARNI group, 13.7% of the patients had severe mitral regurgitation at baseline, which reduced to 7.4% at the 12th month (*P* = 0.058). Compared to ACEI/ARB, there was a trend for improving severe mitral regurgitation by ARNI (*P*= 0.079).

**Table 3:** Follow–up changes in echocardiography in the original cohort.

	**ARNI group**				**ACEi/ARB group**				
**Parameter**	**Valid *N***	**Baseline**	**Follow–up**	**Variation (%)**	**Valid *N***	**Baseline**	**Follow–up**	**Variation (%)**	***P*-value^†^**
LVEF%	105	29.1 ± 6.6	37.4 ± 14.7^*^	8.3 ± 14.6	467	31.7 ± 7.1	42.5 ± 15.7^*^	10.8 ± 15.2	0.228
LVEDD mm	106	60.5 ± 9.2	57.3 ± 8.6^*^	−3.1 ± 7.8	467	56.7 ± 9.0	55.7 ± 8.9^*^	−1.0 ± 6.1	0.013
LVESD mm	106	51.0 ± 8.7	46.3 ± 10.3^*^	−4.7 ± 9.7	467	45.3 ± 10.2	43.2 ± 10.7^*^	−2.1 ± 8.1	0.017
LA mm	107	45.3 ± 8.2	44.5 ± 7.8	−0.8 ± 7.8	469	43.8 ± 7.2	43.5 ± 8.1	−0.2 ± 6.2	0.456
	**Valid** ***N***	**Baseline**	**12 months**	***P*** **-value**	**Valid** ***N***	**Baseline**	**12 months**	***P*** **-value**	
Severe MR	111	13 (13.7%)	7 (7.4%)	0.058	492	25 (7.3%)	25 (7.3%)	1.000	0.079

*ARNI, angiotensin receptor–neprilysin inhibitor; ACEi, angiotensin–converting enzyme inhibitor; ARB, angiotensin receptor blocker; LVEF, center ventricular ejection fraction; LVEDD, center ventricular end–diastolic dimension; LVESD, center ventricular end–systolic diameter; LA, center atrium; MR, mitral regurgitation*.

* *P < 0.05 vs. the baseline value;*

† *The difference in the change between the ARNI and ACEi/ARB groups*.

## Discussion

Data regarding real-world use of ARNI in HFrEF patients with advanced CKD (eGFR ≤ 30 mL/min/1.73 m^2^) is limited; a population that was not included in the PARADIGM-HF trial. In the present study, we found that: (1) the burden of comorbidities was noticeably high (especially DM) in this specific population; (2) the incidence rates of mortality, HF hospitalization, and progression to ESRD were high within 1 year; (3) ARNI and ACEi/ARB users had comparable clinical and renal outcomes; (4) in short-term, ARNIs may be associated with a higher risk of HF hospitalization, especially in patients on dialysis; (5) reverse remodeling was observed in both groups.

In a previous study by our group which also investigated HFrEF patients using the CGRD (regardless of renal function), (7) all-cause mortality occurred in 3.3% and HF hospitalization occurred in 20.8% of the patients within 12 months. In the present study, rates of both all-cause mortality (12.1%) and HF hospitalization (33.8%) within 12 months were much higher. This finding is in concordance with existing evidence, showing that CKD has a negative prognostic impact on patients with HFrEF (1). Moreover, one cohort study reported that 15.3% of patients with stage 4 CKD (46.9% had CV disease, including HF) started renal replacement therapy during an average 23.4 months of follow-up (11). In our study, a similar proportion (14.7%) of the patients progressed to ESRD requiring hemodialysis within only 1 year. These findings highlight the difficulty in caring for patients with both HFrEF and advanced CKD, and that collaborative efforts of both cardiologists and nephrologists are important.

Randomized trials comparing the clinical outcomes of ARNIs with ACEIs/ARBs in patients with HFrEF and advanced CKD are still lacking. One single center observational study showed that patients with stage 4 or 5 CKD treated with ARNI had 28% fewer cardiovascular deaths or HF hospitalizations than those treated with standard HF treatment after a mean follow-up of 15 months, including 102 patients with eGFR of <30 mL/min/1.73 m^2.^. However, the authors did not adjust for confounding factors and there were only 36 patients in the ARNI group and 66 patients in the ACEI/ARB group (12). In a single arm observational study including 23 participants with ESRD on dialysis, ARNI reduced cardiac biomarkers and improved LVEF (13). Hypotension is a well-known adverse effect of ARNI. In the PARADIGM-HF trial,^5^ symptomatic hypotension during randomized treatment occurred more frequently in the sacubitril/valsartan group than in the enalapril group. In the United Kingdom Heart and Renal Protection-III (UK HARP-III) trial which enrolled patients with CKD (eGFR 20 to 60 mL/min/1.73 m^2^), (14) both systolic and diastolic blood pressures were lower in the sacubitril/valsartan group than in the irbesartan group. In persons with advanced CKD, hypotension may lead to renal hypoperfusion, reduced glomerular filtration, and subsequent congestion, which could be a plausible explanation for the higher risk of HF hospitalization in the ARNI group. The risk of HF hospitalization increased shortly (3 months) after the initiation of ARNI and more hyperkalemia in the ARNI group in our study maybe indirect support for this assumption. In dialysis-dependent patients, low blood pressure may result in inadequate fluid removal or even fluid supplement during dialysis. Fluid overload and subsequent acute decompensation can occur as a consequence of inadequate fluid removal during consecutive hemodialysis sessions. Unfortunately, follow-up blood pressure measurements, hypotension episodes, or information regarding net volume removed during dialysis were not available. In summary, the interaction between reverse remodeling, cardiac output, renal perfusion, and medication dosage are complex in this special population. Thus, the appropriate BP thresholds remained to be defined to preserve kidney function while optimizing medical therapies for HFrEF. Also, Future prospectively study with longer follow-up period is needed to illustrate if ARNI is beneficial in persons with HFrEF and severe CKD if blood pressure is periodically monitored, so the dose could be meticulously adjusted.

In our study, reverse remodeling was numerically more pronounced in the ARNI group. In a meta-analysis, (15) ARNI improved left ventricular size and hypertrophy compared with ACEI/ARB in patients with HFrEF, even after short-term follow-up. In a small randomized trial, (16) ARNI reduced mitral regurgitation to a greater extent than did valsartan among patients with functional mitral regurgitation. Reverse remodeling was also observed in persons with HFrEF and ESRD on dialysis in the study by Lee et al. (13). Although the etiology of mitral regurgitation (degenerative or functional) was unavailable in the present study and only half of the patients had follow-up echo, our findings regarding ARNI in reverse remodeling was generally comparable to previous studies.

Since evidence-based pharmacological therapies for persons with both HFrEF and advanced CKD are limited, preventing the development of either disease is the most important task for clinicians. DM is one of the most important upstream risk factors for both HFrEF and CKD. The prevalence of DM has often been reported to be around 35–40% in previous randomized trials or registries of patients with HFrEF (17–19). However, up to 60.2% of the patients had DM in our study. The cardiovascular outcome trials of sodium-glucose cotransporter 2 inhibitors (SGLT2Is) have demonstrated that SGLT2Is can reduce future HF in persons with diabetes (20–22). SGLT2is were also showed to reduce renal events and to slow renal function deterioration in participants with or without diabetes in randomized trials (23, 24) In patients with HFrEF, SLGT2Is slowed the rate of decline in eGFR (25–27). Moreover, in patients with CKD, SGLT2Is reduced the risk of incident HF hospitalization (23, 24) In summary, SGLT2Is should be the first-line treatment for patients with DM, HFrEF, or CKD.

There are several limitations to the present study, First, number of the patients in the ARNI group was small and the follow-up period was short. Second, this was a retrospective observational study. Although we used IPTW to adjust for important outcome-related baseline characteristics, unmeasured confounders may still have been present (including functional class, duration of heart failure, etiology of CKD and HFrEF). Third, missing laboratory data at baseline (such as B-type natriuretic peptide) and the need to input missing values is not uncommon in real-world data and should be acknowledged as another limitation. Clinical events that occurred outside CGMHs were not recorded in the CGRD, which may have led to underestimation of the actual event rates. Forth, this study was conducted using an on-treatment design and did not adjust for temporal changes in medical condition during the follow-up period. Finally, the present study only enrolled Asian patients, and whether our results can be extrapolated to patients of other ethnicities remains unclear.

## Conclusion

Compared to ACEIs or ARBs, ARNIs were associated with comparable clinical and renal outcomes in patients with HFrEF and advanced CKD (eGFR ≤ 30 mL/min/1.73 m^2^). In short-term, HF hospitalization may occur more frequently among ARNI users, especially in patients with ESRD on dialysis.

## Data Availability Statement

The datasets presented in this article are not readily available because the datasets used in this study are available only in the Chang Gung Medical Data Center of Taiwan. Requests to access the datasets should be directed to P-HC, taipei.chu@gmail.com.

## Ethics Statement

The studies involving human participants were reviewed and approved by Chang Gung Memorial Hospital, Link. Written informed consent for participation was not required for this study in accordance with the national legislation and the institutional requirements.

## Author Contributions

F-CH is the guarantor of this work. F-CH, P-HC, and C-CY conceptualized and designed the study. C-PL, C-CY, and Y-CT acquired, analyzed, and interpreted the data. F-CH and C-PL wrote the first draft of the manuscript. C-CY and P-HC drafted the revision. All authors are fully responsible for all content and editorial decisions. All authors have approved the final manuscript.

## Funding

This work was supported by the grants from the Chang Gung Memorial Hospital (CMRPG3K1061). The study funder was not involved in the design of the study, the collection, analysis and interpretation of data, writing the report, and did not impose any restrictions regarding the publication of the report.

## Conflict of Interest

The authors declare that the research was conducted in the absence of any commercial or financial relationships that could be construed as a potential conflict of interest.

## Publisher's Note

All claims expressed in this article are solely those of the authors and do not necessarily represent those of their affiliated organizations, or those of the publisher, the editors and the reviewers. Any product that may be evaluated in this article, or claim that may be made by its manufacturer, is not guaranteed or endorsed by the publisher.
