# Supplementary material for: Angiotensin Receptor-Neprilysin Inhibitors in Patients With Heart Failure With Reduced Ejection Fraction and Advanced Chronic Kidney Disease: A Retrospective Multi-Institutional Study
Source: Front Cardiovasc Med. 2022 Mar 8;9:794707. doi: 10.3389/fcvm.2022.794707 (PMC8963957; doi:10.3389/fcvm.2022.794707)
Supplement: Supplementary file 1 [file Data_Sheet_1.docx]

Supplementary Material

**Supplemental Table 1**. Follow-up outcomes of patients between the ARNI and ACEi/ARBs groups at 12 months of follow up in the propensity score matched cohort

|  | **Data before PSM** | |  | **Data after PSM** | | | | |
| --- | --- | --- | --- | --- | --- | --- | --- | --- |
|  | **ARNI** | **ACEI/ARB** |  | **ARNI** | **ACEI/ARB** |  | **ARNI *vs*. ACEI/ARB** | |
| **Outcome variable** |  |  |  |  |  |  | **HR/SHR (95% CI)** | ***P* value** |
| Patient numbers | 206 | 833 |  | 188 | 188 |  |  |  |
| Primary outcome: composite of heart failure hospitalization and all-cause death | 94 (45.6) | 308 (37.0) |  | 86 (45.7) | 69 (36.7) |  | 1.28 (0.93, 1.76) | 0.124 |
| Secondary outcome |  |  |  |  |  |  |  |  |
| All-cause death | 20 (9.7) | 106 (12.7) |  | 19 (10.1) | 20 (10.6) |  | 0.85 (0.45, 1.61) | 0.623 |
| Heart failure hospitalization | 88 (42.7) | 263 (31.6) |  | 80 (42.6) | 63 (33.5) |  | 1.31 (0.93, 1.83) | 0.122 |
|  |  |  |  |  |  |  |  |  |
| Patient numbers with non-dialysis at baseline | 96 | 325 |  | 84 | 84 |  |  |  |
| Progression to ESRD | 17 (17.7) | 45 (13.8) |  | 15 (17.9) | 7 (8.3) |  | 1.79 (0.76, 4.23) | 0.187 |
| K ≥ 6 mg/dL | 17 (17.7) | 41 (12.6) |  | 14 (16.7) | 7 (8.3) |  | 1.75 (0.68, 4.51) | 0.249 |

ARNI, angiotensin receptor-neprilysin inhibitor; ACEi, angiotensin-converting enzyme inhibitor; ARB, angiotensin receptor blocker; CI, confidence interval; ESRD, end stage renal disease; HR, hazard ratio; PSM, propensity score matching; SHR, subdistribution hazard ratio

**Supplemental Table 2**. Follow-up outcomes of patients without dialysis at baseline between the ARNI and ACEi/ARBs groups at 12 months of follow up after IPTW adjustment

|  | **Data before IPTW** | |  | **Data after IPTW** | | | | |
| --- | --- | --- | --- | --- | --- | --- | --- | --- |
|  | **ARNI**  **(*n* = 96)** | **ACEi/ARB**  **(*n* = 325)** |  | **ARNI** | **ACEi/ARB** |  | **ARNI *vs*. ACEi/ARB** | |
| **Outcome variable** |  |  |  |  |  |  | **HR/SHR (95% CI)** | ***P* value** |
| Composite of heart failure hospitalization and cardiovascular death | 43 (44.8) | 134 (41.2) |  | 43.7% | 41.0% |  | 0.97 (0.61, 1.55) | 0.911 |
| Composite of heart failure hospitalization and all-cause death | 44 (45.8) | 139 (42.8) |  | 44.2% | 43.6% |  | 0.92 (0.58, 1.46) | 0.735 |
| Secondary outcome |  |  |  |  |  |  |  |  |
| All-cause death | 12 (12.5) | 44 (13.5) |  | 17.5% | 14.4% |  | 0.90 (0.45, 1.79) | 0.757 |
| Cardiovascular death | 10 (10.4) | 37 (11.4) |  | 15.9% | 11.2% |  | 1.05 (0.51, 2.16) | 0.905 |
| Heart failure hospitalization | 41 (42.7) | 127 (39.1) |  | 41.5% | 39.2% |  | 0.97 (0.60, 1.55) | 0.893 |
| Admission due to any cause | 51 (53.1) | 172 (52.9) |  | 56.3% | 53.5% |  | 0.95 (0.64, 1.41) | 0.806 |

IPTW, inverse probability of treatment weighting; ARNI, angiotensin receptor-neprilysin inhibitor; ACEi, angiotensin-converting enzyme inhibitor; ARB, angiotensin receptor blocker; HR, hazard ratio; SHR, subdistribution hazard ratio; CI, confidence interval
